# Highly Sensitive Pseudocapacitive Iontronic Pressure Sensor with Broad Sensing Range

**DOI:** 10.1007/s40820-021-00664-w

**Published:** 2021-06-11

**Authors:** Libo Gao, Meng Wang, Weidong Wang, Hongcheng Xu, Yuejiao Wang, Haitao Zhao, Ke Cao, Dandan Xu, Lei Li

**Affiliations:** 1 School of Mechano-Electronic Engineering, Xidian University, Xian, 710071 Shaanxi P. R. China; 2 CityU-Xidian Joint Laboratory of Micro/Nano-Manufacturing, Shenzhen, 518057 P. R. China; 3 Department of Mechanical Engineering, City University of Hong Kong, Kowloon, 999077 Hong Kong SAR P. R. China; 4 Materials Interfaces Center, Shenzhen Institutes of Advanced Technology, Chinese Academy of Sciences, Shenzhen, 518055 Guangdong P. R. China; 5 State Key Laboratory for Mechanical Behavior of Materials, Xian Jiaotong University, No. 28, Xianning West Road, Xian, 710049 Shaanxi P. R. China

**Keywords:** Iontronic sensor, Flexible electronics, Pressure sensor, Pseudocapacitance, Ti_3_C_2_T_x_ MXene

## Abstract

**Highlights:**

- The iontronic pressure sensor achieved an ultrahigh sensitivity (*S*_min_ > 200 kPa^−1^, *S*_max_ > 45,000 kPa^−1^).
- The iontronic pressure sensor exhibited a broad sensing range of over 1.4 MPa.
- Pseudocapacitive iontronic pressure sensor using MXene was proposed.

**ABSTRACT:**

Flexible pressure sensors are unprecedentedly studied on monitoring human physical activities and robotics. Simultaneously, improving the response sensitivity and sensing range of flexible pressure sensors is a great challenge, which hinders the devices’ practical application. Targeting this obstacle, we developed a Ti_3_C_2_T_x_-derived iontronic pressure sensor (TIPS) by taking the advantages of the high intercalation pseudocapacitance under high pressure and rationally designed structural configuration. TIPS achieved an ultrahigh sensitivity (*S*_min_ > 200 kPa^−1^, *S*_max_ > 45,000 kPa^−1^) in a broad sensing range of over 1.4 MPa and low limit of detection of 20 Pa as well as stable long-term working durability for 10,000 cycles. The practical application of TIPS in physical activity monitoring and flexible robot manifested its versatile potential. This study provides a demonstration for exploring pseudocapacitive materials for building flexible iontronic sensors with ultrahigh sensitivity and sensing range to advance the development of high-performance wearable electronics.

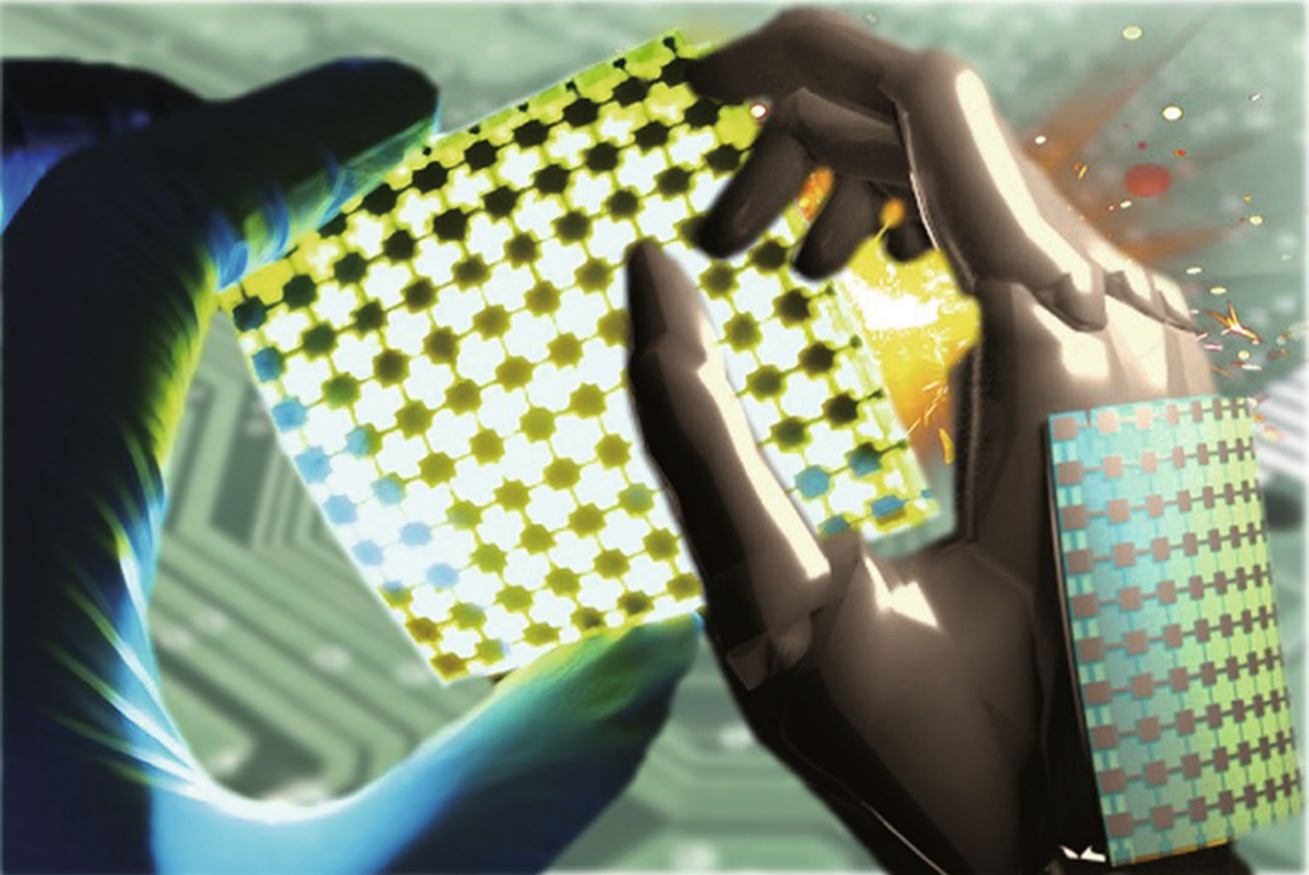

**Supplementary Information:**

The online version contains supplementary material available at 10.1007/s40820-021-00664-w.

## Introduction

The booming development of human–machine interaction (HMI), 5G communication, robotics, wearable electronics and Internet of things (IoTs) has greatly stimulated the demand for wearable sensors [1–10]. Among them, the flexible pressure sensor with high sensitivity and broad sensing range is highly pursued for their good sensing performance to mechanical stimuli along with their ability to be stretched, bent, or twisted into any format under various application scenarios [11–17]. The pressure sensors typically can be classified into four types of piezoresistive, piezocapacitive, piezoelectric, and triboelectricity based on their sensing mechanisms. Piezocapacitive pressure sensor (PPS) has attracted considerable attention due to their merits of simple configuration, low power consumption and favorable working stability [11, 18]. Until now most of the reports so far have demonstrated limited performance, especially the sensitivity (below 10 kPa^−1^) and sensing range (below 1 MPa) [19–21]. Therefore, improving the sensitivity and sensing range of PPS is necessary to further extend the reach of existing applications.

Several strategies were developed to improve the sensitivity and sensing range of the PPS, such as (a) building microstructures on electrode or dielectric; (b) using a composite dielectric; (c) engineering holes inside the dielectric layer [22]. Engineering microstructure of the electrode or dielectric is widely used. For instance, Guo et al. achieved a graded intrafillable architecture-based iontronic PPS with unprecedently high sensitivity (*S*_min_ > 220 kPa^−1^) and a broad pressure regime (0.08 Pa–360 kPa) [23]. Kim et al. developed the top floating electrode for iontronic PPS with greatly enhanced sensitivity [20]. Additionally, recently introducing the remarkable interfacial capacitance for PPS by electrical double layer (EDL) effect is also an effective strategy [24]. For example, Pan et al. introduced the iontronic PPS based on the EDL capacitive materials [21, 25, 26], such as graphene [20], silver [21], and Au [23]. Thanks to its high areal capacitance (up to nF or even near µF), the EDL-based iontronic sensor showed much higher sensitivity and broader sensing range than traditional PPS. While both of these two strategies have been greatly improved the devices’ sensitivity and sensing range, there is still plenty of room for performance improvement. For example, the idea that using pseudocapacitive electrode materials instead of EDL materials can enhance the capacitance has already been demonstrated in electrochemical supercapacitor devices [27]. As one might expect, using pseudocapacitive iontronic PPS combined with a novelly designed configuration would enhance the sensitivity and sensing range significantly.

Herein, we developed a Ti_3_C_2_T_x_-derived iontronic pressure sensor (TIPS) with a novel configuration of the top floating electrode along with a microstructured dielectric film. Ti_3_C_2_T_x_ has superior electronic conductivity (~10,000 S cm^−1^), electrochemical, optical and mechanical properties. It presents a wide range of application in energy storage, sensor, electromagnetic interference and optoelectronics. It was chosen as electrodes of flexible PPS by using its ions intercalation-based pseudocapacitance that significantly exceeds EDL electrodes materials. The floating electrode design along with the microstructured dielectric film further enhanced the sensitivity deeply. Benefited from the synergetic effect between the electrode materials and the device configuration, TIPS exhibited unprecedentedly ultrahigh sensitivity (*S*_min_ > 200 kPa^−1^, *S*_max_ > 45,000 kPa^−1^), broad sensing range (20 Pa to 1.4 MPa), low limit of detection (LOD) of 20 Pa as well as stable long-term working durability for 10,000 cycles. The sensors are capable of monitoring the physical activity and flexible robot tactile perception. Additionally, the electrode patterns were fabricated via laser-engraving technologies, indicating the manufacturing scalability. This work developed here shed light on the advance of PPS.

## Experimental Sections

### Preparation of the MXene (Ti_3_C_2_T_x_) Nanosheets Solution

The preparation method is similar to reports elsewhere [28]. Ti_3_C_2_T_x_ was prepared by immersing 1 g of Ti_3_AlC_2_ in 40 mL of a hydrochloric acid solution containing 1.6 g of LiF and 9 M HCl at 35 ℃ for 24 h. In this process, HF selectively etched the aluminum in Ti_3_AlC_2_. The obtained multilayer Ti_3_C_2_T_x_ suspension was centrifuged at 3500 rpm for 5 min to obtain the precipitate, which was rinsed with deionized water until the pH value of the solution was larger than 6. The precipitate was then dispersed in deionized (DI) water and continuously ultrasonicated for 3 h under the protection atmosphere of argon. Finally, the suspension was centrifuged at 3500 rpm for 60 min to obtain a dark colloidal Ti_3_C_2_Tx nanosheets solution.

### Preparation of the Ti_3_C_2_T_x_ Electrode

The n-WF was washed with alcohol and DI water for three times, respectively. The dried n-WF was directly immersed into the Ti_3_C_2_Tx nanosheets solution (5 mg mL^−1^) for 10 s and pulled out slowly for drying at 50 °C. To find the optimal amount of MXene for n-WF, this dip-coating process was repeated for 4 times. A thin gold layer, acting as the current collector, was deposited on the conductive n-WF using the magnetic sputtering for 1 min. The obtained conductive n-WF composite material was carefully laminated on PI/PET or pure PI film. The laser (power: 7 W, speed: 2000 mm min^−1^) was used to engrave the designed pattern. The designed electrode was obtained on flexible substrate after peeling off the unused part.

### Preparation of the Flexible Sensor

PVA-KOH was chosen as the dielectric membrane in the sensor. The preparation of the PVA-KOH was modified from the reported method reported in our previous studies [29, 30]. PVA-KOH gel electrolyte was directly spin-coated on the abrasive paper (400#) and dried at 50 °C for 0.5 h. The flexible and mechanically robust film (20 µm) can be easily peeled off from the abrasive paper. The ionic membrane was closely attached to the bottom electrode using a soft Ecoflex silicone film with a cavity as an air spacer. To assemble the device, a PET or PI was used to cover the device by double faced adhesive tape (3 M) to bond them together tightly for testing and application study.

### Structural Characterization

Field emission SEM (FESEM, Quanta 450, 20 kV) and the transmission electron microscope (TEM, JEM 2100F) equipped with energy-dispersive spectroscopy (EDS) were used to characterize the structural and morphology information. X-ray diffractometer (XRD, Bruker, D8 ADVANCE) with Cu Kα radiation (*λ* = 0.15406 nm) was used to identify the phase structure.

### Mechanical and Electromechanical Characterizations

The mechanical performance of the n-WF and ionic membrane was conducted on a mechanical testing machine (Zhi Qu, 990B) with a pulling speed of 0.5 mm min^−1^. For each testing condition, 5 samples were repeated, and the final result was obtained by averaging. For the electromechanical characterization, the variable resistance, current and capacitance can be recorded in this process by the electrochemical working station (CHI 760E), LCR impedance analyzer (Tonghui, TH 2827A) and IM3536 LCR meter. The CV curves also were recorded by the electrochemical working station under the applied pressure.

### Application of the Sensor

The customized soft gripper can easily be fabricated by 3D printing method. The flexible sensor was mounted onto the end of the gripper and the gripper was fixed onto a three-axis mobile platform and can move freely. The whole system was controlled through wireless remote-control method, which can be control by a micro controller (STM32), and the corresponding signal was captured by an LCR impedance analyzer.

## Results and Discussion

### Device Fabrication and Structural Characterization

**Fig. 1:**
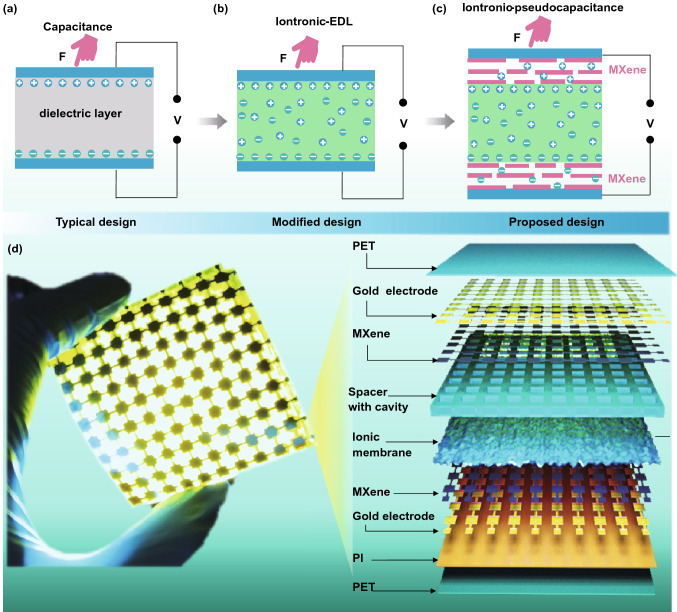
Flexible TIPS layout and design. Schematic illustration of the mechanism of the **a** traditional PPS, **b** modified design and **c** proposed mechanism of our pseudocapacitance-derived sensors. **d** Digital optical image of the TIPS arrays under bending state and corresponding exploded view layout of the various layers in the sensor

The traditional parallel-plate capacitive sensor is typically composed of a dielectric layer sandwiched between two conducting electrode plates. Its corresponding capacitance was defined as *C* = *ε*_0_*ε*_r_*A*/*d*, where *ε*_0_ and *ε*_r_ are the permittivity of vacuum and dielectric layer, *A* is the overlapped area, and *d* is the distance between the two electrode plates, respectively (Fig. 1a). The measured capacitance value of such type of device is typically in the pF-level, which result in inferior sensitivities and increased difficulties in the subsequent circuit processing of the signal. The iontronic sensor is therefore designed in order to increase its capacitance. The previously reported iontronic sensors were merely adopted to the EDL-based electrode materials, hindering their capacitance (Fig. 1b). Here, for the first time, MXene was employed as the electrode materials in the iontronic sensor, which has a high capacitance compared to the EDL-based electrode (Fig. 1c) [31, 32]. The contribution of MXene capacitance is primarily derived from the bottom and upper electrode layer by K^+^ intercalating into Ti_3_C_2_T_x_, resulting in superior sensitivity (*S*) according to *S* = Δ*C*/*C*_0_, where Δ*C* is the capacitance variation and *C*_0_ is the initial capacitance [33]. Figure 1d shows the as-prepared bendable, flexible sensor array device, which was mainly composed of Ti_3_C_2_T_x_ electrodes coated with the gold layer (current collector), ionic membrane (20 µm) with rough a surface and spacer (500 µm in thickness) with cavity. This rational structure design contributes to a significantly enhance in sensitivity because the initial capacitance is much lower than that under pressure, as direct contact between the upper electrode and the ionic membrane was blocked by the spacer initially. Once the electrode touched the ionic membrane is induced by the applied pressure, the capacitance would dramatically increase due to the shortened pathway between the electrodes.

To obtain the desired electrode pattern, we directly used the laser to engrave Ti_3_C_2_T_x_ composite materials laminated on a polyethylene terephthalate (PET)/polyimide (PI)-laminated film (~120 µm). Here, the laser-scribing technique with higher pixel fill factor was used to precisely pattern the electrode arrays (10 × 10 in 5 × 5 cm^2^, Fig. S1a and Experimental Sections). On the other hand, the dielectric layer, another core part of capacitive sensor device, played an important role in enhancing the sensitivity, detection limit and sensing range. To further boost the sensor’s behavior, geometric microengineering design of the dielectric layer was adopted as it can increase the capacitance variation at a given applied pressure through enhancing the electrode distance change and effective dielectric constant [34]. In this study, we directly spin-coated polyvinyl alcohol (PVA)-KOH gel on rough abrasive paper as a mold. Then, the translucent and mechanically flexible film can be easily peeled off from the mold (Fig. S1b and Experimental Sections).

**Fig. 2:**
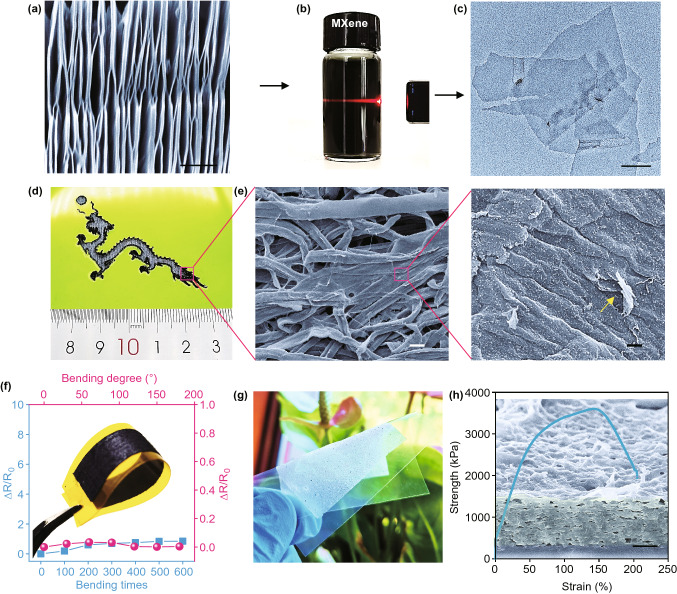
Structural characterization of the sensing materials and electrode. **a** SEM image of the accordion-microstructured MXene. The scale bar is 500 nm. **b** Tyndall effect of the Ti_3_C_2_T_x_ nanosheets solution. **c** TEM image of the Ti_3_C_2_T_x_ nanosheets. The scale bar is 100 nm. **d** Laser pattern of a Chinese dragon. **e** SEM image and its corresponding large view of the Ti_3_C_2_T_x_ nanosheets coated n-WF. The scale bar is 40 µm and 500 nm, respectively. **f** Bending deformation of the Ti_3_C_2_T_x_ electrode and its corresponding resistance change. Inset is the optical image of the Ti_3_C_2_T_x_ -coated n-WF. **g** Iontronic film on a bending PET film. **h** Mechanical performance of the PVA-KOH film. Inset is corresponding cross-sectional view of the PVA-KOH film. The scale bar is 10 µm

To prepare the Ti_3_C_2_T_x_ electrode, corresponding Ti_3_C_2_T_x_ nanosheets were firstly synthesized by chemically etching the Al elements in Ti_3_AlC_2_ MAX phase with smooth surfaces and stacked layers (see Experimental Sections and Figs. S2, S3) [31, 35]. Figure 2a shows the accordion-microstructured Ti_3_C_2_T_x_. In the X-ray diffraction (XRD) patterns of the as-synthesized product, a strong (002) peak located at 6.8° (corresponding d-spacing is 1.2 nm) demonstrates the successful removal of Ti–Al bond and the formation of Ti_3_C_2_T_x_ (Fig. S4) [36]. The typical Tyndall effect further evidenced the highly homogeneous distribution of Ti_3_C_2_T_x_ nanosheets in water. This is due to the abundantly existed hydroxide and fluorine groups, which is beneficial to the uniform coating of Ti_3_C_2_T_x_ layer on the substrate (Fig. 2b) [37]. The as-obtained Ti_3_C_2_T_x_ nanosheets exhibited 2D features with size of 300–500 nm (Fig. 2c). Then, a simple yet effective “dip-coating” strategy was used to fabricate the soft and robust electrode building on the nonwoven fabrics (n-WF). Benefited from the flexibility and high resolution (~100 µm) of the laser-scribing strategy, complicated fabrics patterns, such as the Chinese dragon, can be achieved (Fig. 2d). The SEM images suggested the uniform and thin coating of Ti_3_C_2_T_x_ nanosheets on the raw n-WF, which possessed a coarse surface formed by intertwined and tangled fibers, along with abundant porosities within the weaved electrode compared to the raw n-WF (Figs. 2e and S5). The geometrical entanglement of the individual fiber contributed to enhance the mechanical performance and conductive stability of the composites. The increased roughness results in a larger accessible surface area and thus improved the electrochemical performance for high specific capacitance accordingly. Additionally, the performances of the composite materials are largely dependent on the dip-coating times. Figure S6a–e shows the morphology of the pristine n-WFs and samples coated for 1–4 times, respectively. With the increased coating times, the sheet resistance decreased from 4.6 to 0.9 Ω cm^−2^ (Fig. S6f), yet the Ti_3_C_2_T_x_ nanosheets seriously aggregated together when coating reached 4 times; therefore, the n-WF was dip-coated 3 times in the following study. The mechanical performance of the MXene-modified n-WF was also studied and showed a good performance compared with pure n-WF, with merely a slight mechanical decay (Fig. S7). Specifically, the resistance variation of the composite materials was below 1.5 times below the initial value. It kept stable from 200 to 600 cycles under a bending degree of 90° as the fiber structure reached a stable condition. Similarly, the sample’s conductivity also possessed a steady-state even under bending from 0 to 180° (Fig. 2f).

Additionally, as shown in Figs. S8 and 2g, h, the translucent ionic film maintained a structural integrity under bending deformation on a flexible PET substrate and has successfully replicated the random rough microstructures of the abrasive paper, which increased the sensitivity of the sensor in this study for the following reasons. Firstly, the permittivity of dielectric layer changes when the sensor is compressed, because the displaced volume is air, in which the permittivity is usually lower than that of elastomers [23]. Secondly, the distance between the electrode and the ion membrane reduces greatly, leading to a shortened charge transfer distance, whereas the contact area increased for promoting electrochemical reactions; both contribute in improving the capacitance. Moreover, the tensile test of the film demonstrated an excellent stretchability as large as 150% and fracture strength of 3.5 MPa, which is necessary for building the flexible electronics.

### Device Characterization

**Fig. 3:**
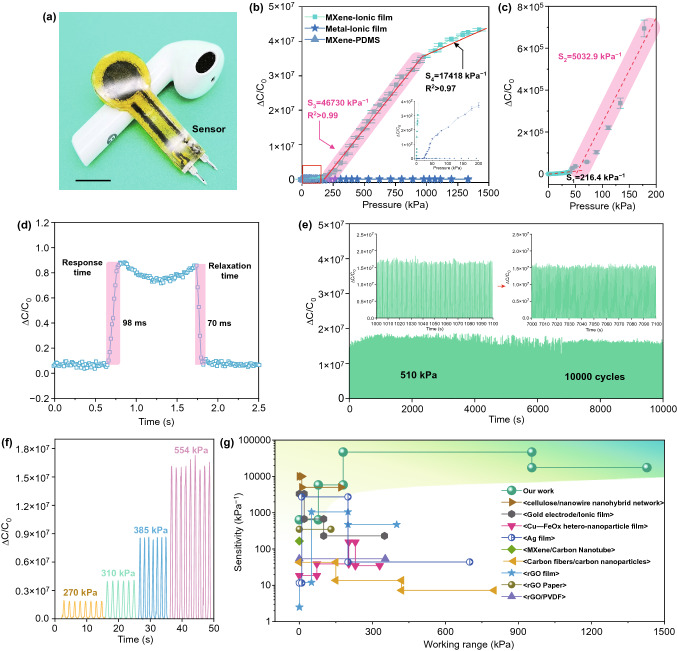
Characterization of the sensor. **a** Digital optical image of the single sensor device. **b** Capacitance variation versus the pressure change of the sensing materials and **c** its corresponding large view. **d** Response and relaxation time of the flexible sensor. **e** Long-time durability of the sensor under 510 kPa. **f** Capacitance variation of the sensor under various pressure. **g** Comparison of our sensor’s sensitivity and sensing range with other reported work

The sensitivity, LOD, pressure sensing range, response and relaxation time and the long-term durability are studied to evaluate the sensor’s performance. Figure 3a shows a digital optical image of a single sensor. Figure 3b shows the normalized capacitance variation of the sensors under different pressures. The TIPS showed an ultrahigh sensitivity in a broader pressure sensing range compared to the traditional ones. It had a high sensitivity up to 46,730 kPa^−1^ within the pressure range of 200–800 kPa, which is much higher than the control sensors of MXene-PDMS, Metal-ionic-based counterparts, and even the TIPS without the spacer (Fig. S9). At high-pressure regime, TIPS still showed superior sensitivity of 17,148 kPa^−1^ up to 1.4 MPa. Even at relatively low-pressure range, the sensor exhibited outstanding sensitivities over 200 and 5000 kPa^−1^ in the range below 60 and 200 kPa, respectively (Fig. 3c). These results are almost the best one among the reported sensitivity, with the broadest sensing range for the flexible capacitive sensor (Table S1). The response time and relaxation time of TIPS are only 98 and 70 ms, respectively, demonstrating that the device responds quickly to the external pressure (Fig. 3d). The long-term durability test of TIPS was studied under pressure of 510 kPa (Fig. 3e). A robust signal output without any decay was observed during 10,000 cycles. It is ascribed to the electromechanical robustness of the n-WF MXene electrode and ionic membrane of the sensor (Fig. 2f–h). The electromechanical behaviors of TIPS under various dynamic pressures (270, 310, 285 and 554 kPa) were studied. It showed a stable and proportional electrical signal at different pressures (Fig. 3f). The LOD for the sensor is only 20 Pa, indicating the great sensitivity to the tiny force (Fig. S10). Figure 3g shows the sensitivity of the sensor relative to the reported works. The developed sensor not only showed an ultrahigh sensitivity, but also an incomparably broad pressure sensing range up to 1.4 MPa. Its performance is superior to the existing pressure sensors [23, 38–45]. To sum up, the iontronic sensor presents superior sensitivity, sensing range, LOD, response/relaxation time when compared to other similar iontronic works (Table S1).

**Fig. 4:**
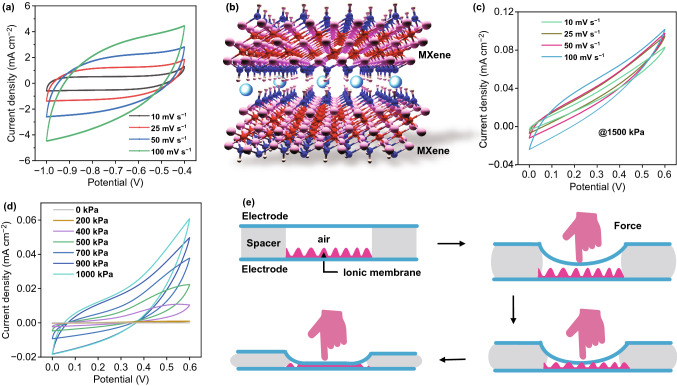
Mechanism of the iontronic sensor. **a** CV curves of the MXene tested in three-electrode mode in KOH solution. **b** Intercalation of K^+^ between Ti_3_C_2_T_x_ layers **c** CV curves of the symmetrical configuration of the MXene electrode materials tested in two-electrode mode at various scan rates under applied pressure of 1500 kPa. **d** CV curves of the symmetrical configuration of the MXene electrode materials tested in two-electrode mode at various applied pressures. **e** Schematic illustration of the working mechanism of the iontronic sensor

To gain further insight into Ti_3_C_2_T_x_ capacitive behavior and elucidate its sensing mechanism, electrochemical evaluations based on both three-electrode system and two-electrode system were carried out. As shown in Fig. 4a, the rectangular shape of the cyclic voltammetry (CV) curves of Ti_3_C_2_T_x_ appeared in a three-electrode system in the 2 M KOH electrolyte, demonstrating the typical capacitive behavior through the intercalation of K^+^ and H_2_O molecules between the Ti_3_C_2_T_x_ layers (Fig. 4b) [33, 46, 47]. Considering that the iontronic sensor can also be regarded as the symmetrical supercapacitor device under the applied pressure, its performance in the two-electrode system was also studied. There was no obvious sluggish observed in the curves, further illustrating the capacitive behaviors of the sensor at various scan rates from 10 to 100 mV s^−1^ under 1500 kPa (Fig. 4c). The shape of the device CV curves would be changed under different pressure since increasing applied pressure would results in capacitance enhancement (Fig. 4d). As expected, the profile of the device CV changed accordingly to the variation of applied pressure from unloaded state of 0 to 1000 kPa. It should be mentioned that the capacitance is too low to be exhibited at CV curves under its original state due to the isolation of the electrode and ionic membrane (Fig. S11a) [21]. The CV curves under various applied pressures from 200 to 1000 kPa exhibited different integrated areas; it is because that with higher pressure, it is more likely to enable improved access areas and short transportation pathway to deep trap sites induced by larger contact areas. The integrated area showed almost linear behaviors, which are in accordance with the results above (Fig. S11b). Based on the above discussion, the sensing process is mainly composed of the following stages (Fig. 4e). Initially, TIPS worked as a normal PPS with a low capacitance due to the separation of Ti_3_C_2_T_x_ electrodes and ionic membrane caused by the air spacer. With the increased applied pressure, the capacitance increases due to the reduced distance between two electrodes. Once the applied pressure increased to a state where the upper electrode and ionic membrane were compressed to start contacting with each other through the holes of air spacer, the capacitance increased instantly as the ionic transportation occurred. It is worth to mention that the thickness, modulus of the spacer played an important role in the sensing performance (more information can be found in Figs. S12 and S13 [26]*.* We therefore used the soft silicone with 500 µm as the spacer to set the pressure threshold (444.4 Pa in this study). Additionally, the microstructure and drying state of the ionic film also very important as shown in Figs. S14–S16. Once the contacting started, the sensor still has a lot of room to increase capacitance under added pressure, resulting from the low contact surface area at the beginning and rough surface (duplicating from the abrasive paper) of the membrane. Further adding pressure would promote an intimate contact of the upper/bottom electrode with the membrane and thus lead to an ultrahigh capacitance due to the Ti_3_C_2_T_x_-induced intercalating behaviors. The Ti_3_C_2_T_x_-induced ultrahigh pseudocapacitance, compare to the typical EDL capacitance, results in the high capacitance variation. However, considered that the mechanical property of the substrate materials used, the sensing range therefore is determined to be 1.4 MPa (Fig. S7). Throughout the process, the flexible yet mechanically robust n-WF with constructed intertwined structure provided an electrode substrate with largely enhanced surface area, which contributes to the excellent electrochemical performance. Besides, the intertwined and porous layer is expected to prevent the restacking of flakes and facilitate ionic transport and access to Ti_3_C_2_T_x_ nanosheets. Overall, the spacer determined a dominant role in the sensitivity, while the pseudocapacitance also made a great contribution, because of its significant decrease of the initial capacitance to increase the sensitivity. Such a rationally designed flexible pressure sensor with ultrahigh sensitivity and broader sensing range, using the MXene as the capacitive materials, opens a gate for more promising applications.

### Application of the Sensor

**Fig. 5:**
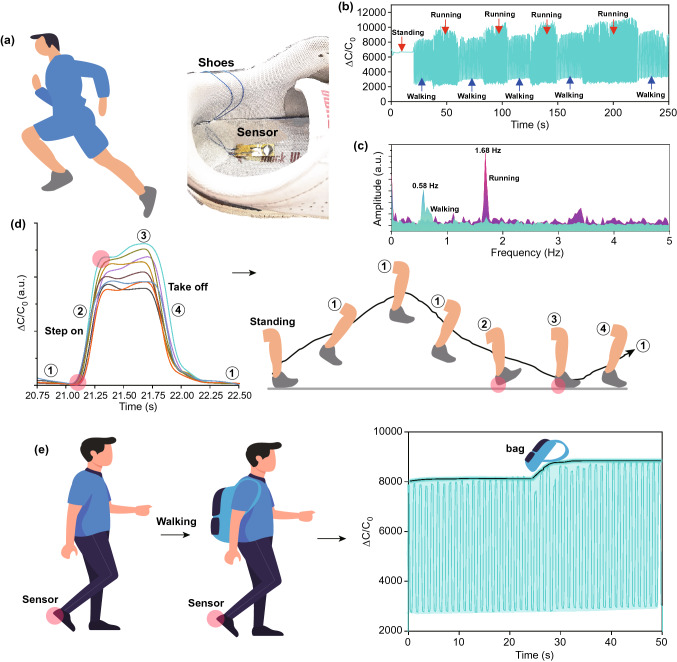
Practical application of the flexible iontronic sensor on monitoring of physical activity. **a** Optical image of the sensor mounted into the shoes to monitor the physical activity. **b** Physical activity such as standing, walking and running were monitored by the sensor. **c** The FFT spectrum of the walking and running obtained from (**b**). **d** Walking state was analyzed by the flexible sensor. **e** Sensor’s sensitivity on monitoring the walking statues with different bearing load

The practical applications of the sensor are shown in Fig. 5. Firstly, the sensing performance of our sensor was evaluated via detecting foot plantar pressure. The sensor was mounted inside the shoes, where high pressure forms during running or strenuous exercise caused by the rapid change of the acceleration (Fig. 5a). The activities of a human’s standing, walking and running was successfully monitored (Fig. 5b). The fast Fourier transform (FFT) results indicate that both the intensity and frequency of the output signal for running state are larger than those of walking state (Fig. 5c). Data of the sensor for walking process of the sensor were collected to analyze the physical activity. It was clearly observed that the profiles remained a similar tendency (Fig. 5d). It was mainly composed of 4 processes. From the step I to step II, the pressure gradually increased during the compaction of the feet sole and the ground and then reached its maximum value upon the foot sole in full contact with the ground (III) and finally decreased to its original state I with the foot lifted (IV). Even for a long-term walking, the sensor can keep a reliable stability (Fig. S17). Additionally, we also tested the force resolution of the TIPS to monitor the bear load for a man (Body weight, 71 kg). During the test, the sensor mounted in a shoe is capable of accurately detecting the added load (one bag, 1 kg) for a man under walking state, demonstrating its potential application on a smart insole system [48]. The application of the sensor at low pressure was also investigated as shown in Fig. S18, which can successfully monitor the pulse rate and eyes blink. Even in a harsh environment, the sensor enabled to retain its original capacitance for a long-term durability (Fig. S19).

**Fig. 6:**
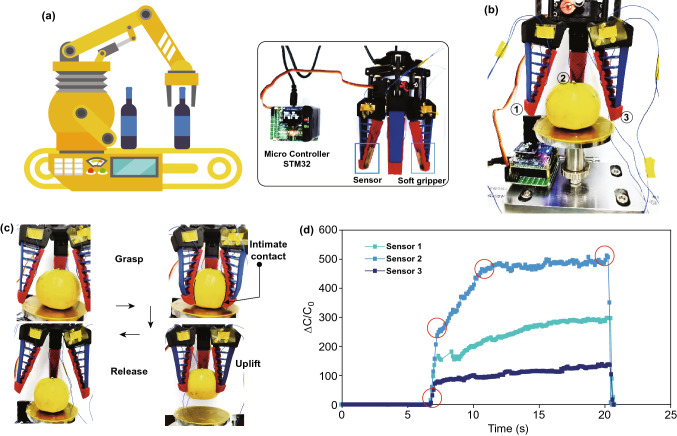
Practical application of the flexible iontronic sensor for electronic skin. **a**, **b** Configuration of the wirelessly controlled soft gripper with flexible sensor to achieve product grasping. **c** Procedures for the soft gripper to capture and release the orange. **d** Capacitance variations of the sensor during the process to grasp and release the sample

To further demonstrate its application as a tactile sensor, we directly installed the sensor on the surface of a customized soft gripper contacting with the grasped objects. The corresponding setup is shown in Fig. 6a. Since the traditional rigid grippers cannot directly capture the objects with irregular shapes or soft properties due to lack of conformality, a customized soft fin ray-like manipulator was designed and fabricated. The gripper was controlled to grasp an orange, and the corresponding grasping force was monitored by the sensor during this process (Fig. 6b, c). It can be seen that the soft gripper possessed perfect shape adaptability to fit the geometrical morphology of the orange and successfully moved it up. The force initially increased rapidly in grasping the orange, then slowly increased when lifting it up, and finally reached a stable level to fully fix the orange. Once releasing the orange, the force decreased instantly to its initial value. Finally, a sensor array was practically demonstrated to recognize the geometric projection shape of various samples including flash disk and solid gum with a concave bottom, indicating its high resolution and large-scale manufacturing potential (Fig. S20).

## Conclusion

In summary, Ti_3_C_2_T_x_ is used as the pseudocapacitive electrode materials to fabricate the flexible iontronic sensor instead of the energy storage device for the first time. Due to the intercalation-induced high capacitance and rationally designed structural configuration, the sensor showed unprecedentedly ultrahigh sensitivity of over 46,730 kPa^−1^ and incomparable broad sensing range of 1.4 MPa with long-term durability (no decay over 10,000 cycles) under high pressure. Specifically, the high-pressure results in an increase in the accessible surface area and shortened ions transportation pathway for the MXene-based sensor. Additionally, a facile laser-engraving was proved to be a fast methodology for the composite electrode, which can also be extended to fabricate other MXene-based composite structures in the future for flexible electronics. As practical demonstrations, the flexible sensor not only can precisely distinguish the exercise frequency and the intensity when monitoring the human activities with attached to the shoe sole, but also can be used as a pressure-sensing skin for measuring the grasping force of a soft robotic gripper. This study rationally exploited MXene’s intercalation-induced pseudocapacitance for the iontronic pressure sensor beyond typically developing the MXene as energy storage materials or simply using its conductive feature as a piezoresistive sensor. We believe that our work will significantly broaden the research and application of the iontronic sensor through using the pseudocapacitive or even battery-type capacitive materials instead of the traditional EDL electrode. This work gives a concept guide for the experts to explore more potential in supercapacitor area.

## Supplementary Information

Below is the link to the electronic supplementary material.

Supplementary file1 (PDF 1162 kb)
