# Supplementary material for: Highly Sensitive Pseudocapacitive Iontronic Pressure Sensor with Broad Sensing Range
Source: Nanomicro Lett. 2021 Jun 11;13:140. doi: 10.1007/s40820-021-00664-w (PMC8193410; doi:10.1007/s40820-021-00664-w)
Supplement: Supplementary file 1 — Supplementary file1 (PDF 1162 kb) [file 40820_2021_664_MOESM1_ESM.pdf]

Supporting Information for

Highly Sensitive Pseudocapacitive Iontronic Pressure Sensor with Broad Sensing Range

Libo Gao[1, 2, *], Meng Wang[1, 2], Weidong Wang[1, 2, *], Hongcheng Xu[1, 2], Yuejiao Wang[3], Haitao Zhao[4], Ke Cao[1, 2], Dandan Xu[1, 2], Lei Li[5, *]

[1]School of Mechano-Electronic Engineering, Xidian University, Xi'an 710071 Shaanxi, P. R. China

[2]CityU-Xidian Joint Laboratory of Micro/Nano-Manufacturing, Shenzhen 518057, P. R. China

[3]Department of Mechanical Engineering, City University of Hong Kong, Kowloon 999077, Hong Kong SAR, P. R. China

[4]Materials Interfaces Center, Shenzhen Institutes of Advanced Technology, Chinese Academy of Sciences, Shenzhen 518055, Guangdong, P. R. China

[5]State Key Laboratory for Mechanical Behavior of Materials, Xi'an Jiaotong University, No. 28, Xianning West Road, Xi'an, 710049 Shaanxi, P. R. China

*Corresponding authors. E-mail: lbgao@xidian.edu.cn (Libo Gao); wangwd@mail.xidian.edu.cn (Weidong Wang); l-li@xjtu.edu.cn (Lei Li)

Supplementary Tables and Figures

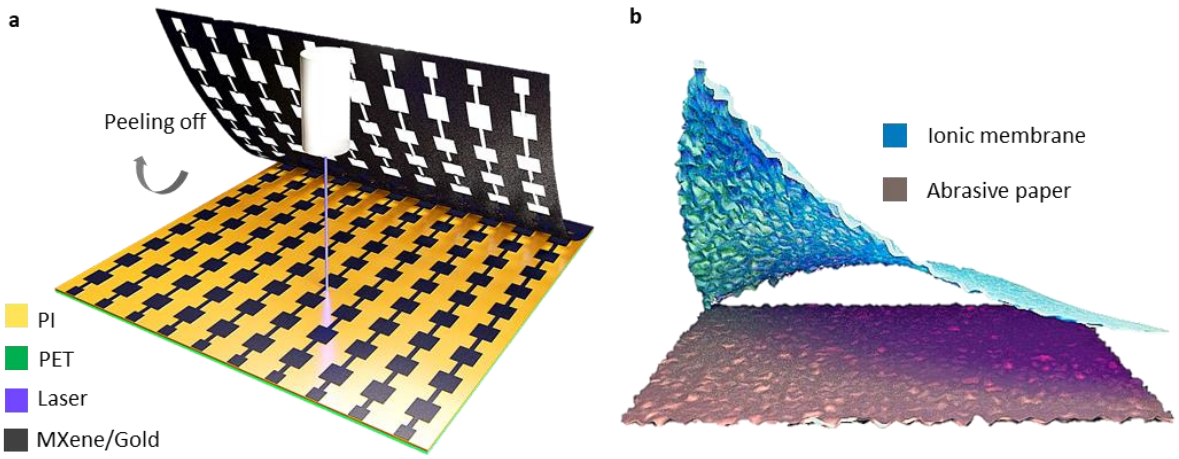

Fig. S1 (**a**) Using laser engraving to fabricate the $Ti_3C_2T_x$ electrode pattern on PET/PI film. (**b**) Schematic illustration of preparation of the ionic membrane on abrasive paper

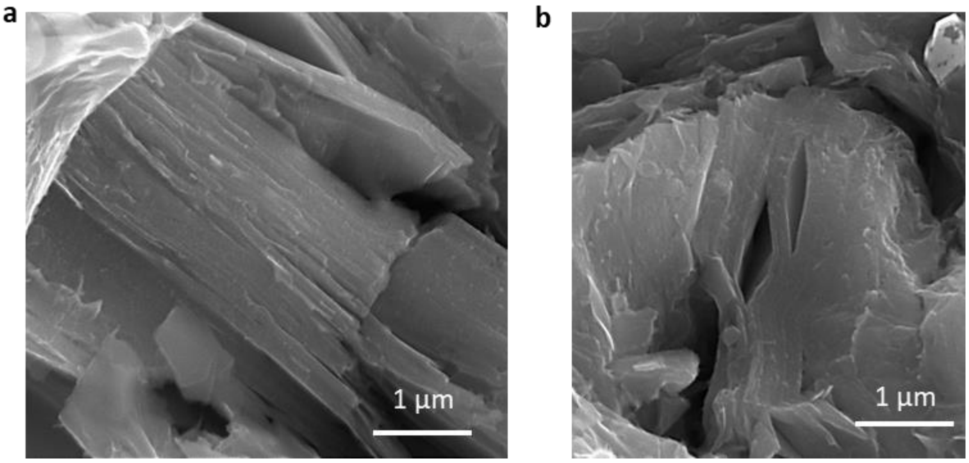

Fig. S2 (**a-b**) Scanning electron microscope (SEM) of Ti_3AlC_2 MAX

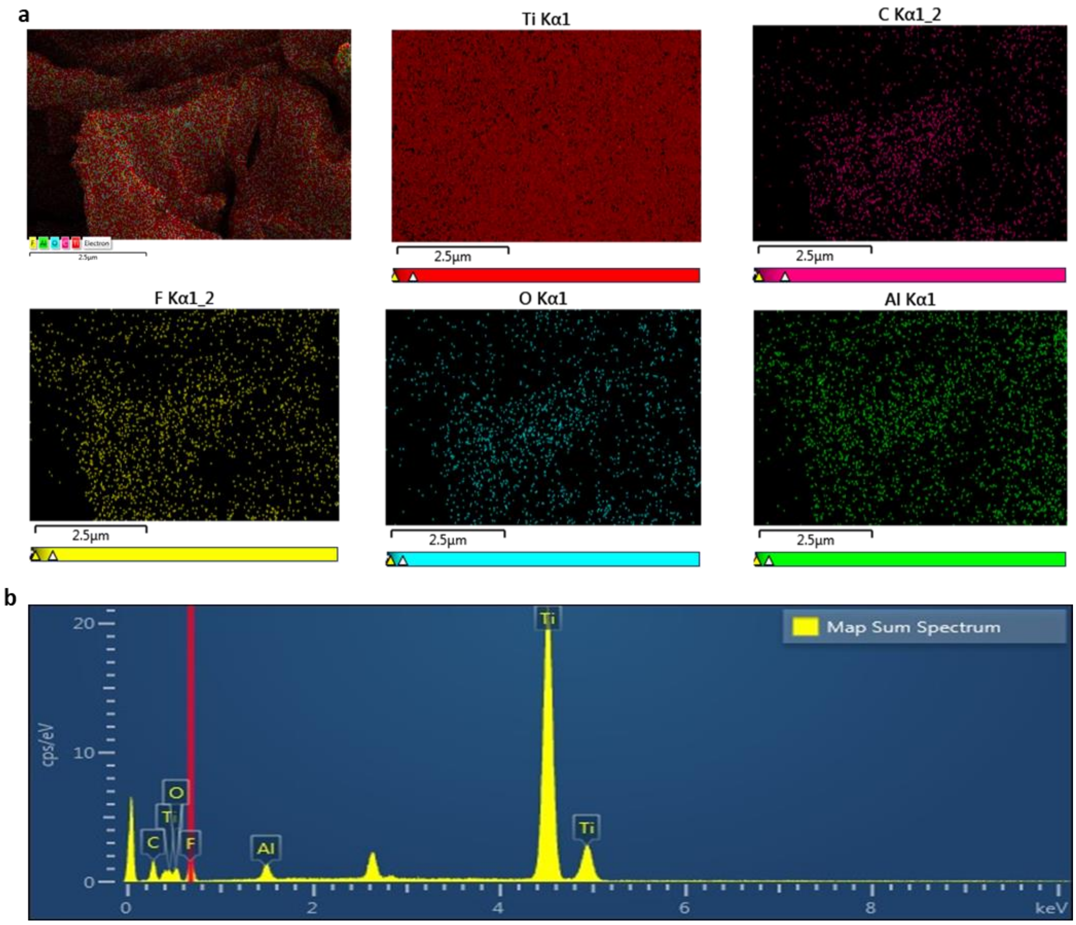

Fig. S3 SEM-energy dispersive spectroscopy (EDS) of MAX phase. (**a**) Element mapping distribution of MAX. (**b**) Corresponding element content of MAX

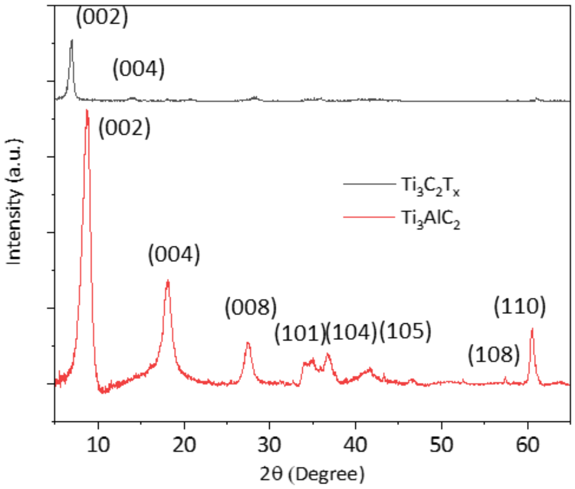

Fig. S4 X-ray diffractometer (XRD) of $Ti_3C_2T_X$ and Ti_3AlC_2

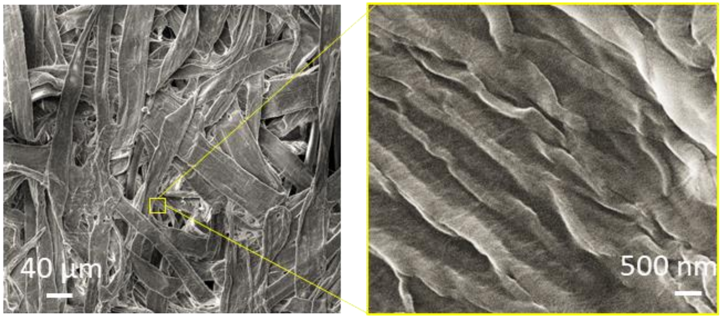

Fig. S5 SEM image and its corresponding large view of the non-woven fabrics (n-WF). The scale bar is 40 μm and 500 nm

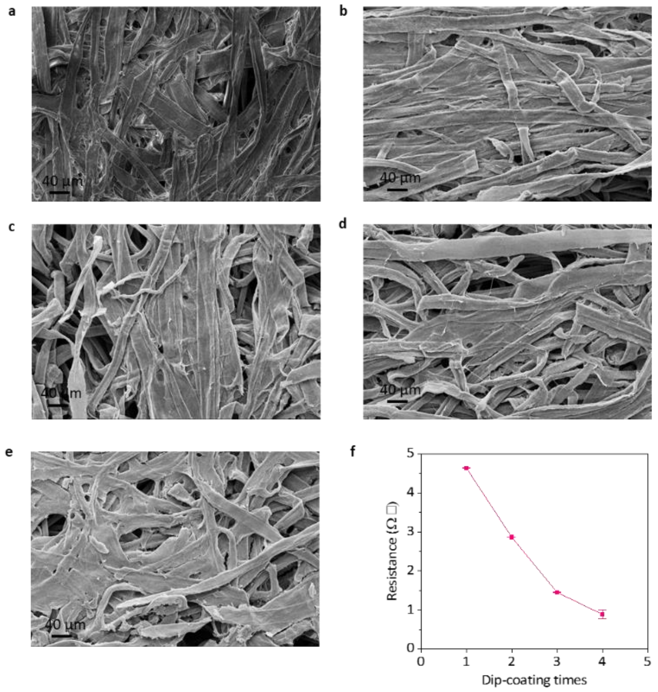

Fig. S6 SEM image of the n-WF dipped into MXene solution for (**a**) 0, (**b**) 1, (**c**)2, (**d**)3, (**e**) 4 times, respectively. (**f**) The corresponding resistance with the dip-coating times of n-WF

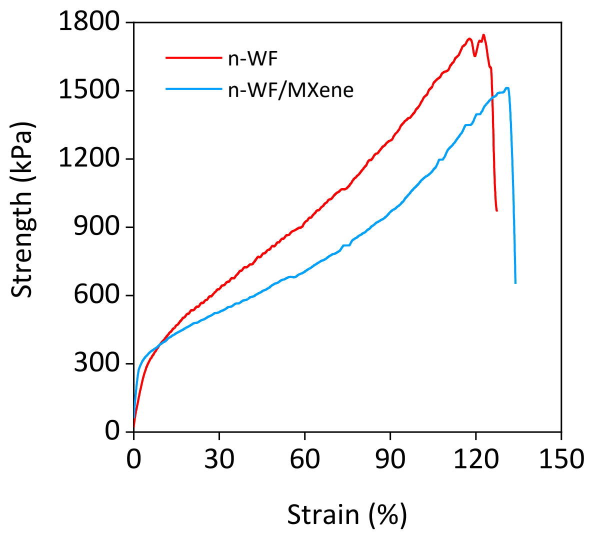

Fig. S7 Tensile test of the n-WF and n-WF/MXene

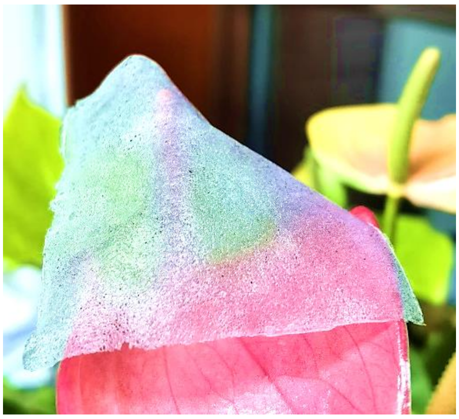

Fig. S8 The translucent and mechanically flexible PVA-KOH membrane

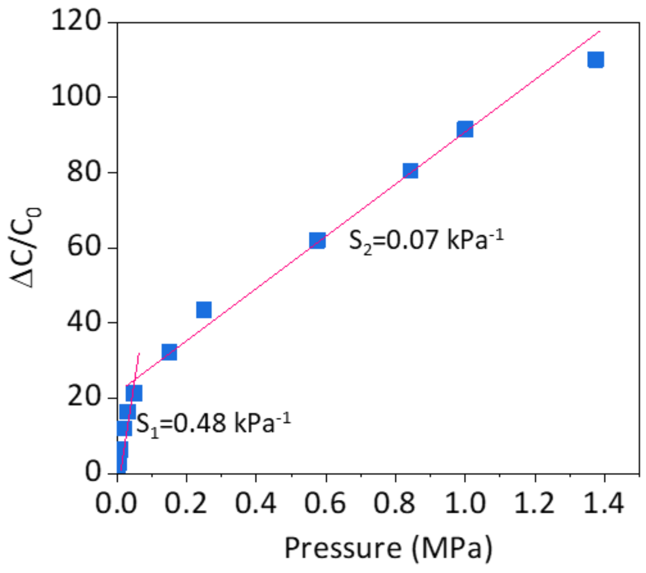

Fig. S9 Capacitance variation of the iontronic sensor without spacer

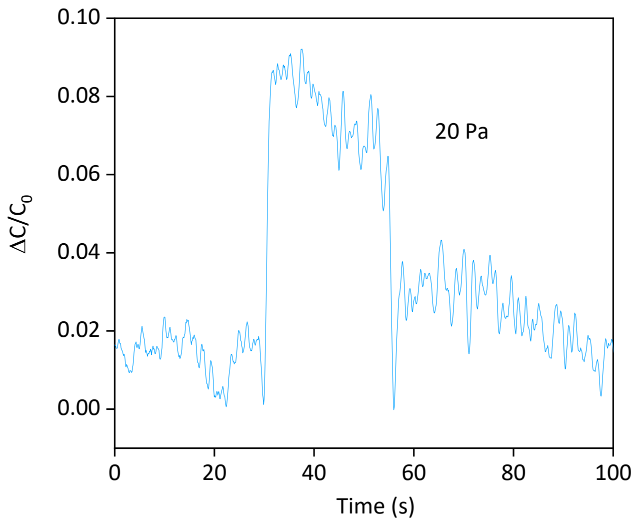

Fig. S10 Capacitance variation of the iontronic sensor under an applied pressure of 20 Pa

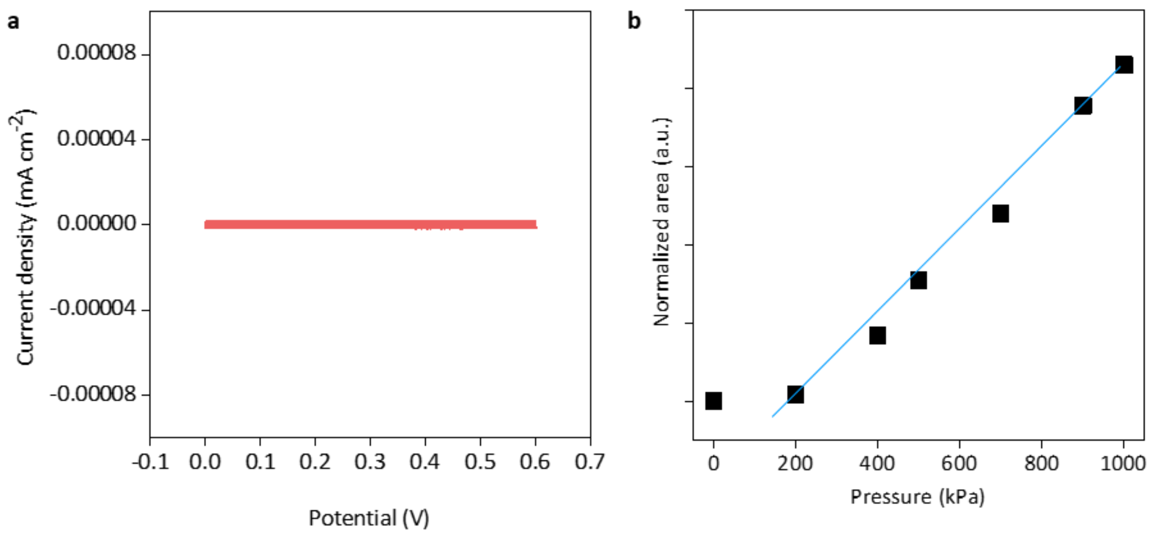

Fig. S11 Electrochemical tests of the symmetrical supercapacitor. (**a**) CV curves of the symmetrical configuration of MXene electrode materials tested in two-electrode mode at 0 kPa. (**b**) Normalized integrated areas of the CV curves of the device under various pressure

Firstly, the thickness cannot be too thin, or the electrode would easily touch the ionic film during the device assembly process. In our study, we found that the 500 μm is optimal which can effectively prevent the touching while keep the high sensitivity. Actually the thickness of the spacer's performance on the sensor has already be carefully analyzed by Pan et al. [S1] And in their work, the thinner spacer would lead to a high sensitivity and low detection limit, especially at low sensing range. However, the sensing range would be limited. Similarly, our sensor also showed an inferior low detection limit and sensitivity at low sensing range when a 1000 μm sized spacer was used. Therefore, the spacer in our work is 500 μm.

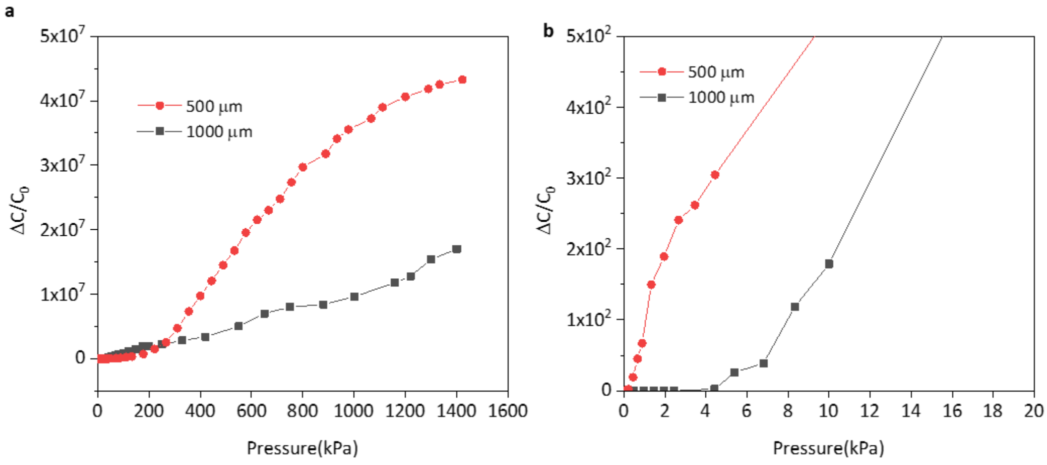

Fig. S12 Capacitance variation of the sensor with various spacer thickness under various applied pressure from (**a**) 0 to 1400 kPa and (**b**) 0 to 20 kPa

The soft materials would lead to a relatively easy deformation which can greatly increase the sensitivity and low detection limit at low sensing range. The spacer materials used in our work is Ecoflex silicone, which is very soft and widely used in flexible electronics. As shown below, the sensor showed a superior low detection limit and sensitivity at low sensing range (below 10 kPa) compared to the PDMS. However, the PDMS-based sensor showed a higher sensitivity compared to that of Ecoflex silicone around the 50-100 kPa range, which because that this range belongs to the threshold of PDMS while the Ecoflex silicone already reached its threshold. Considered the sensitivity in low sensing range, low detection limit and whole sensing range, we choose the Ecoflex silicone as the optimal materials spacer.

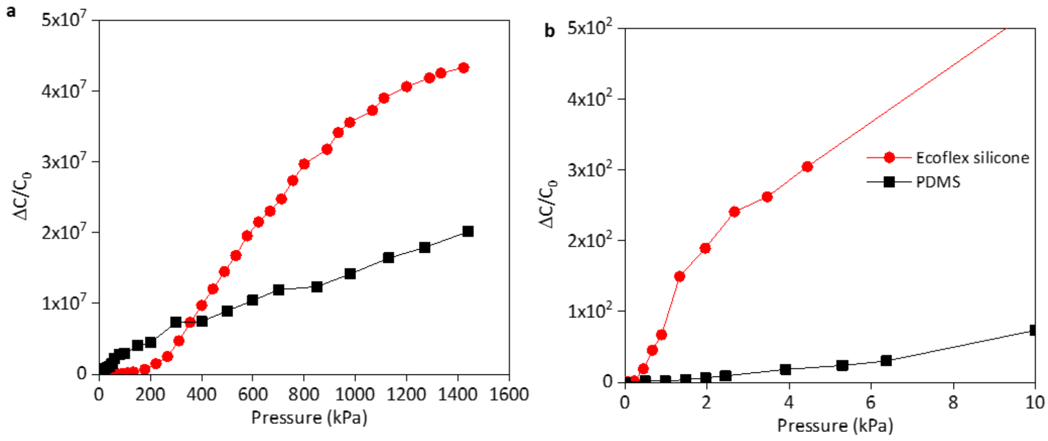

Fig. S13 Capacitance variation of the sensor using different materials as the spacer

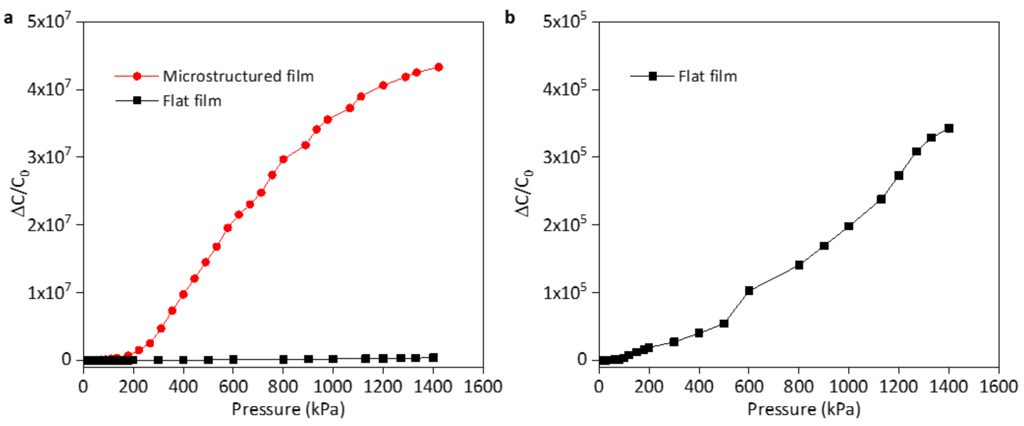

Fig. S14 (**a**) Capacitance variation of the sensor with ionic film with or without microstructure under pressure. (**b**) Capacitance variation of the sensor with flat film

Obviously, the sensor with the ionic film treated for 30 min showed better performance compared to the 45 min- and 60 min-sample, which is due to the fast ions transportation and enhanced areal capacitance.

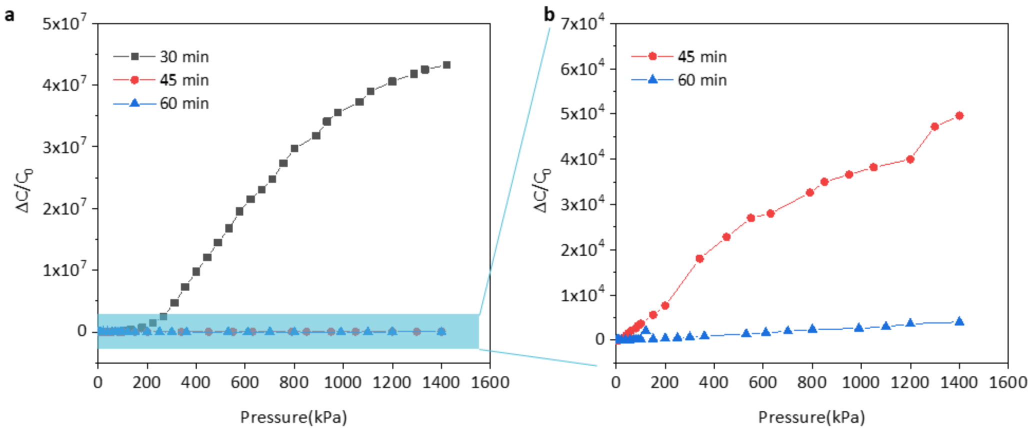

Fig. S15 (**a**) Capacitance variation of the sensor with various ionic film treated by different time and (**b**) corresponding enlarged view

The lifespan of the sensor was also investigated when increasing the drying time. Here we choose the ionic film treated for 45 min as a sample to test its long-term durability. As shown below, the sample still exhibited 82% capacitance retention after10000 cycles under a pressure of 500 kPa. However, the 30 min-sample retain almost no any decay (Figure 3 in main text). Such behavior is mainly ascribed to that the ionic film become fragile with less water content.

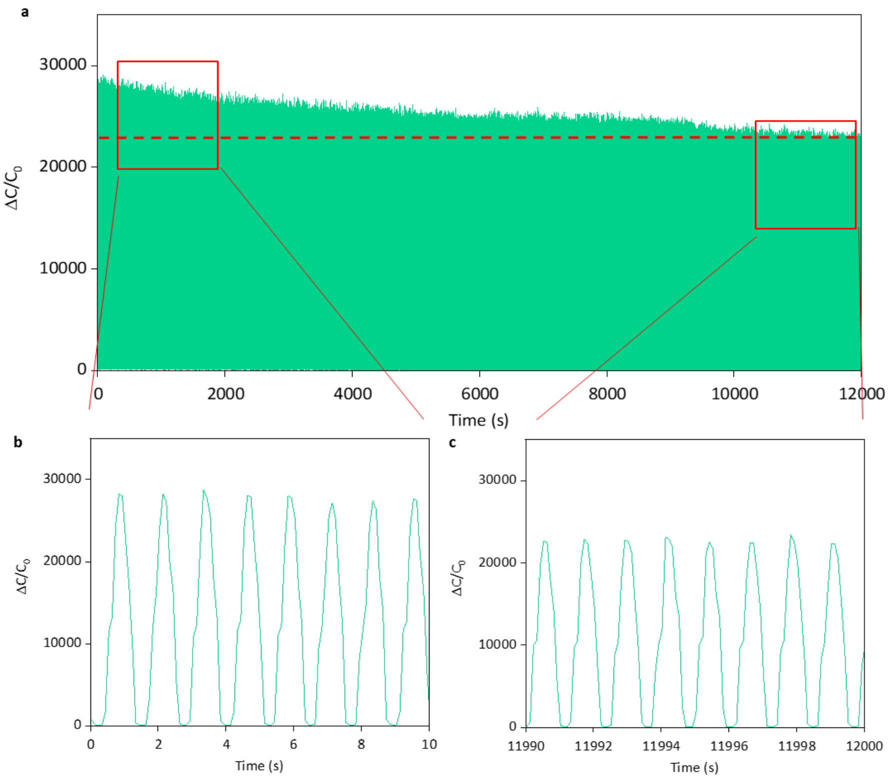

Fig. S16 (**a**) Long-term cycling test and corresponding (**b**) initial (**c**) last cycles of the ionic sensor

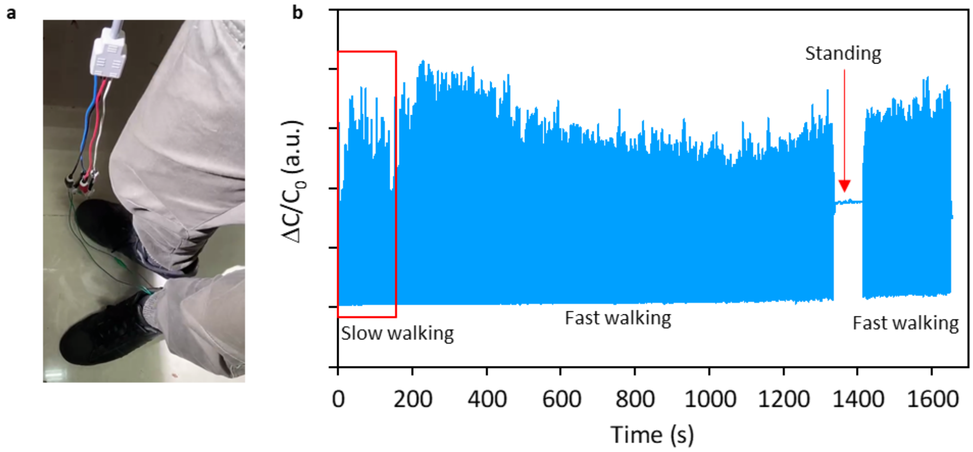

Fig. S17 (**a**) The digital optical image of the sensor used in monitoring of the walking status. (**b**) Corresponding data curve of the capacitance change as a function of the time

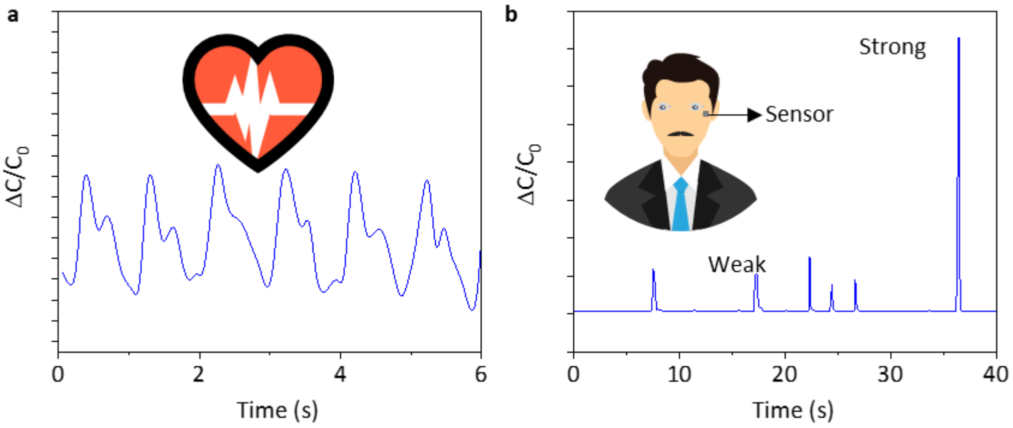

Fig. S18 Low pressure application of the sensor on (**a**) pulse rate and (**b**) eyes blink

The sensor was put into a sealing bottle filled with a saturated NaCl solution which the humidity is 75% RH. As shown below, the initial value rapidly increased when the humidity increased from 25% RH to 75% RH. This increase is still in the acceptable regime when considered its ultrahigh sensitivity used in pressure sensor. Further, the sensor remains a stable value variation in the humidity of 75% RH for a long-term of over 16000 s.

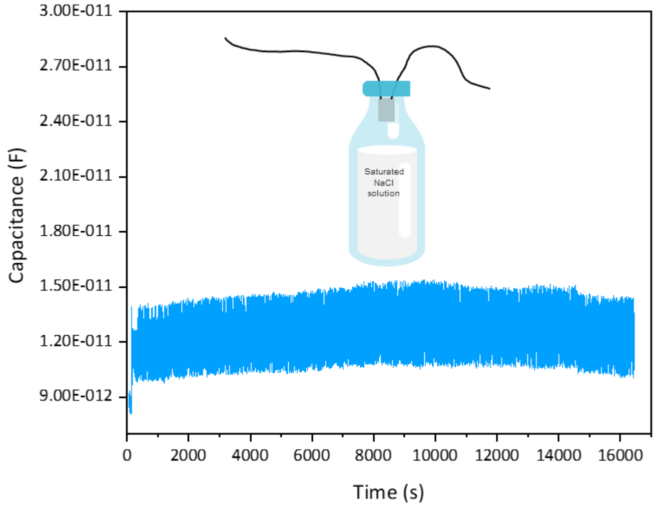

Fig. S19 The capacitance variation of the sensor under 75% RH for a long time

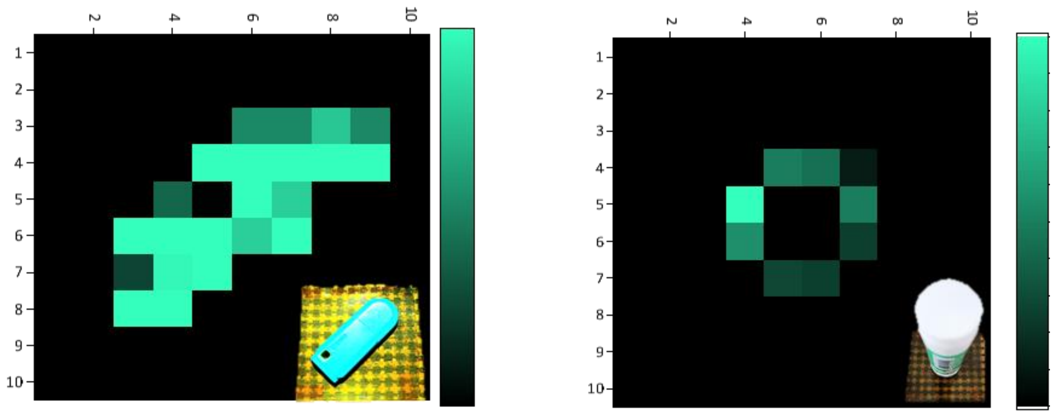

Fig. S20 Flexible sensor arrays could recognize (**a**) the geometric projection shape of flash disk and (**b**) solid gum with concave bottom

Table S1 Comparisons of our work with the reported flexible pressure sensors

No.	Type	Device materials	Highest sensitivity	Working range	LOD	Response/Relaxation time	Refs.
1	Iontronic	Gold electrode /PVA-H_2SO_4	3300 kPa^{-1}	360 kPa	0.08 Pa	9 ms/18 ms	[S2]
2	Iontronic	ITO/Ionic Liquid	0.43 nF kPa^{-1}	~100 kPa	33 Pa	NG	[S3]
3	Iontronic	Silver /Ionic gel film	308 nF kPa^{-1}	4 KPa	NG	272 ms/234 ms	[S4]
4	Iontronic	Ag NWs/PVDF	1.194 kPa^{-1}	120 KPa	0.4 Pa	40 ms/NG	[S5]
5	Iontronic	ITO/ Nafion film	5 nF kPa^{-1}	30 kPa	NG	NG	[S1]
6	Iontronic	AgNWs/ Ionic film	131.2 kPa^{-1}	32.35 kPa	1.12 Pa	43 ms/71 ms	[S6]
7	Iontronic	Graphene/ Ionic Liquid	31.1 kPa^{-1}	15 kPa	NG	100 ms/100 ms	[S7]
8	Piezoresistive	Te-PEDOT:PSS	10000 kPa^{-1}	160 kPa	NG	1 ms	[S8]
9	Piezoresistive	rGO/PDMS	1051 kPa^{-1}	400 kPa	10 Pa	150 ms/40 ms	[S9]
**10**	**Iontronic**	**MXene/PVA-KOH**	**46730 kPa^{-1}**	**1.4 MPa**	**20 Pa**	**98 ms/70 ms**	**Our work**

Note: Low limit of detection (LOD), Polyvinyl alcohol (PVA), Polydimethylsiloxane (PDMS), Ag nanowires (Ag NW)

Supplementary References

[S1] Z. Zhu, R. Li, T. Pan, Imperceptible Epidermal–iontronic interface for wearable sensing. Adv. Mater. **30**, 1–9 (2018). https://doi.org/10.1002/adma.201705122

[S2] N. Bai, L. Wang, Q. Wang, J. Deng, Y. Wang et al., Graded intrafillable

architecture-based iontronic pressure sensor with ultra-broad-range high sensitivity. Nat. Commun. **11**, 3–11 (2020). https://doi.org/10.1038/s41467-019-14054-9

[S3] B. Nie, R. Li, J.D. Brandt, T. Pan, Iontronic microdroplet array for flexible ultrasensitive tactile sensing. Lab Chip **14**, 1107–1116 (2014). https://doi.org/10.1039/c3lc50994j

[S4] K. Keum, J. Eom, J.H. Lee, J.S. Heo, S.K. Park et al., Fully-integrated wearable pressure sensor array enabled by highly sensitive textile-based capacitive ionotronic devices. Nano Energy **79**, 101631 (2021). https://doi.org/10.1016/j.nanoen.2020.105479

[S5] Q. Liu, Z. Liu, C. Li, K. Xie, P. Zhu et al., Highly transparent and flexible iontronic pressure sensors based on an opaque to transparent transition. Adv. Sci. **7**, 2000348 (2020). https://doi.org/10.1002/advs.202000348

[S6] A. Chhetry, J. Kim, H. Yoon, J.Y. Park, Ultrasensitive interfacial capacitive pressure sensor based on a randomly distributed microstructured iontronic film for wearable applications. ACS Appl. Mater. Interfaces **11**, 3438–3449 (2019). https://doi.org/10.1021/acsami.8b17765

[S7] J.S. Kim, S.C. Lee, J. Hwang, E. Lee, K. Cho et al., Enhanced sensitivity of iontronic graphene tactile sensors facilitated by spreading of ionic liquid pinned on graphene grid. Adv. Funct. Mater. **30**, 1908993 (2020). https://doi.org/10.1002/adfm.201908993

[S8] B. Lee, J.Y. Oh, H. Cho, C.W. Joo, H. Yoon et al., Ultraflexible and transparent electroluminescent skin for real-time and super-resolution imaging of pressure distribution. Nat. Commun. **11**, 1–11 (2020). https://doi.org/10.1038/s41467-020-14485-9

[S9] X. Tang, C. Wu, L. Gan, T. Zhang, T. Zhou et al., Multilevel microstructured flexible pressure sensors with ultrahigh sensitivity and ultrawide pressure range for versatile electronic skins. Small **15**, 1–9 (2019). https://doi.org/10.1002/smll.201804559
